# Estimated Cancer Risk in Females Who Meet the Criteria to Exit Cervical Cancer Screening

**DOI:** 10.1001/jamanetworkopen.2025.0479

**Published:** 2025-03-12

**Authors:** Shalini L. Kulasingam, Inge M. C. M. de Kok, Abhinav Mehta, Erik E. L. Jansen, Mary Caroline Regan, James W. Killen, Stephen Sy, Ran Zhao, Karen Canfell, Jane J. Kim, Megan A. Smith, Nicole G. Campos

**Affiliations:** 1 Division of Epidemiology & Community Health, School of Public Health, University of Minnesota, Minneapolis; 2 Erasmus University Medical Center, Rotterdam, the Netherlands; 3 Center for Health Decision Science, Harvard T.H. Chan School of Public Health, Boston, Massachusetts; 4 The Daffodil Centre, University of Sydney, a joint venture with Cancer Council New South Wales, Sydney, Australia

## Abstract

**Importance:**

Cervical screening guidelines in the US recommend that most females can exit routine screening at age 65 years following 2 recent consecutive negative cotest results (concurrent human papillomavirus and cytology tests). However, empirical data on the subsequent risks of cancer and cancer death in this subgroup of females are limited.

**Objective:**

To estimate the risks of cervical cancer and cervical cancer death among females who meet the cotesting criteria to exit screening.

**Design, Setting, and Participants:**

In this decision analytical comparative modeling study, 4 decision analytical models from the Cancer Intervention and Surveillance Modeling Network–Cervical modeling consortium that fit common US epidemiological data targets were validated against published estimates of 3- and 5-year risks of cervical intraepithelial neoplasia grade 3 (CIN3) among females meeting exit criteria at Kaiser Permanente Northern California (KPNC).

**Main Outcomes and Measures:**

Age-conditional and cumulative risks of cervical cancer and cervical cancer death at ages 65, 70, 75, 80, and 85 years were estimated by performing a comparative modeling analysis of the 4 models to estimate the risks of cervical cancer and cervical cancer death after exiting screening.

**Results:**

All models estimated a 5-year risk of CIN3 that was within the range of empirical data from KPNC. Projections of the cumulative and age-conditional risks of cervical cancer and cervical cancer death increased with time since exiting screening. The cumulative risks of cervical cancer and cervical cancer death by age 70 years were estimated to range from 0.001% to 0.003% and from 0% to 0.001%, respectively. The cumulative risks of cervical cancer and cervical cancer death by age 85 years ranged from 0.026% to 0.081% and from 0.005% to 0.038%, respectively, across models. Results were sensitive to assumptions about screening test sensitivity and incidence of high-risk human papillomavirus.

**Conclusions and Relevance:**

In this decision analytical comparative modeling study, a low risk of cervical cancer and cervical cancer death was estimated among females who fulfilled the US criteria to exit screening with cotesting; however, the risks increased with age and/or time since screening exit. The findings suggest that future guidelines should consider acceptable risk levels when defining screening modality and exit age requirements.

## Key Points

**Question:**

What is the risk of cervical cancer and cervical cancer death among females who have met the criteria to exit cervical cancer screening in the US?

**Findings:**

In this decision analytical comparative modeling study, the cumulative risks of cervical cancer and cervical cancer death by age 70 years were estimated to range from 0.001% to 0.003% and from 0% to 0.001%, respectively. By age 85 years, these cumulative risks were estimated to range from 0.026% to 0.081% and from 0.005% to 0.038%, respectively.

**Meaning:**

The findings suggest that the estimated risk of cervical cancer and cervical cancer death in females who have met the criteria to exit screening is low but increases with age.

## Teaser

This decision analytical comparative modeling study estimates risk of cervical cancer and cervical cancer death among females aged 60 years or older who have met US guideline criteria to exit cervical cancer screening.

## Introduction

In 2012, the American Cancer Society, American Society for Colposcopy and Cervical Pathology, American Society for Clinical Pathology, and US Preventive Services Task Force (USPSTF) guidelines were harmonized with a recommendation that females who have been adequately screened for cervical cancer can exit screening at age 65 years.^1,2^ Adequate screening is defined as either 3 consecutive negative Papanicolaou test results (ie, cytology) or 2 consecutive negative concurrent human papillomavirus (HPV) and Papanicolaou test results (ie, cotests) since age 55 years among females, with the most recent test administered within the past 5 years. Although recent updates to the guidelines have occurred, recommendations regarding the criteria and age to end screening have remained unchanged since 2012.^3^ As noted by the USPSTF in its recommendation statement, however, the epidemiologic evidence to support a definitive age to end screening in older females is lacking.^3^

While modeling has been used to estimate the risk of cancer in females who have been screened with cytology or HPV-based tests,^4,5^ to our knowledge, the risk in the subgroup of females who have met the cotesting criteria to exit screening in the US has not been estimated. Until recently, there were limited empirical data to validate model-estimated risks of cervical intraepithelial neoplasia grade 3 (CIN3), a surrogate outcome for cancer risk, and of cancer in well-screened populations. A study by Landy et al^6^ calculated the 3- and 5-year risks of cervical precancer (defined as CIN3 or adenocarcinoma in situ) based on an analysis of over 346 000 females aged 55 to 64 years who attended screening at Kaiser Permanente Northern California (KPNC). For females aged 60 to 64 years at their second consecutive negative cotest result, the 3- and 5-year risks of CIN3 were 0.015% (95% CI, 0%-0.031%) and 0.058% (95% CI, 0.006%-0.109%), respectively. No females in this cohort were diagnosed with cervical cancer during follow-up.

The Cancer Intervention and Surveillance Modeling Network (CISNET) is a National Cancer Institute–funded consortium of investigators that uses independently developed microsimulation models to address research and policy questions related to certain cancers. CISNET–Cervical Cancer (CISNET-CC) includes 4 models of HPV infection and cervical pathogenesis that have been calibrated using US data.^7,8^ Because these models were independently developed, comparative modeling exercises that yield similar conclusions can improve the credibility and robustness of findings. Differences in model results may point to areas of uncertainty and highlight future research needs.

The objectives of the present study were to use the 4 CISNET-CC US models to (1) validate model-estimated 3- and 5-year risks of CIN3 against the observed risks of CIN3 in the KPNC cohort and (2) estimate the age-conditional and cumulative risks of cervical cancer and cervical cancer death up to and including age 85 years among females who cease screening following 2 consecutive negative cotest results between ages 60 and 65 years.

## Methods

### Model Description

In this decision analytical comparative modeling study, 4 CISNET-CC microsimulation models (Harvard; Microsimulation Screening Analysis [MISCAN]–Cervix [Erasmus Medical Center]; Policy1-Cervix [Daffodil Centre]; and University of Minnesota–HPV Cancer [UMN-HPV CA]) were used to estimate risks of CIN3, cervical cancer, and cervical cancer deaths in a cohort of females not vaccinated against HPV. These models are calibrated to a common set of data from empirical studies summarizing the burden and natural history of HPV and cervical cancer in the US but vary in their underlying structure and assumptions of the carcinogenic process (Table 1). Given the use of aggregate secondary data for model parameterization in conjunction with simulation modeling, this analysis was considered non–human participant research and did not require institutional review board review. This study followed the Consolidated Health Economic Evaluation Reporting Standards (CHEERS)^16^ and HPV-FRAME reporting guidelines^17^ when applicable.

**Table 1.** Key Features, Sources of Data, and Assumptions of the 4 CISNET Cervical Carcinogenesis Models

	Harvard	MISCAN-Cervix	UMN-HPV CA Cervix	Policy1-Cervix
**Natural history**				
HPV types modeled	HPV 16, 18, 31, 33, 45, 52, and 58; other high risk; low risk	HPV 16, 18, and pooled 31, 33, 45, 52, and 58; other high risk	HPV 16, 18 and, pooled 31, 33, 45, 52, and 58; other high risk	HPV 16, 18, and pooled 31, 33, 45, 52, and 58; other high risk
States modeled	Normal, HPV, CIN grade 2, CIN grade 3, cancer by stage, cancer death	Normal, HPV, CIN grades 1-3, cancer by stage, cancer death	Normal, HPV, CIN grades 1-3, cancer by stage, cancer death	Normal, HPV, CIN grades 1-3, cancer by stage, cancer death
High-risk HPV prevalence at age ≥65 y, %	5.7	6.9	6.5	5.6
Progression and regression rates for precancer	HPV type and time in state dependent	Age and HPV type dependent	Age and HPV type dependent	Age and HPV type dependent
Cancer stage–specific detection via symptoms per year, %	Early:19; regional: 60; distant: 90	Early: 1-26 (mean, 2.6 y)^a^; regional: 39-63 (mean, 6.0 y)^a^; distant: 100 (mean, 1.3 y)^a^	Early: 15; regional: 23-60; distant: 90	Early: 15; regional: 30; distant: 90
Cancer stage–specific progression, %/y	Early to regional: 11; regional to distant: 26	Early to regional: 74-99 (mean, 2.6 y)^b^; regional to distant: 36-61 (mean, 6.0 y)^b^	Early to regional: 22; regional to distant: 26	Early to regional: 6-65; regional to distant: 45
SEER survival time, y^9^	20	15	10	10
Hysterectomy^10,11^	NHDS	NHDS	NHDS	NHDS
Competing causes of death^12^	Berkeley life tables	Berkeley life tables	Berkeley life tables	Berkeley life tables
Lifetime risk of cancer in the absence of screening, %^c^	1.3	0.9	2.3	1.5
Proportion of risk attributable to cancers detected in females aged 65-85 y, %	26.1	33.0	28.0	27.9
**Screening**				
Cytology^4,5^				
Sensitivity, %^d^	51.4-72.7	42.3-85.8^e^	51.4-72.7	51.4-72.7
Specificity, %	88.0-91.9	82.9-99.4^e^	88.0-91.9	88.0-91.9
HPV^4,5^				
Sensitivity, %^f^	96.0	94.0	87.0-95.0	93.5
Specificity, %	88.0	69.0-100	85.0	93.1^g^
Follow-up of abnormal results^13^	ASCCP	ASCCP	ASCCP	ASCCP
Adherence to colposcopy and biopsy, %^14,15^^h^	78.9-100	78.9-100	78.9-100	78.9-100
Colposcopy and biopsy sensitivity for CIN grade 2 or 3, %	100	100	100	100
Adherence to treatment, %^14,15^^h^	73.4-100	100^i^	73.4-100	73.4-100
Treatment efficacy, %^19^	93	100^i^	93-100	93-100

Abbreviations: ASCCP, American Society for Colposcopy and Cervical Pathology; CIN, cervical intraepithelial neoplasia; CISNET, Cancer Intervention and Surveillance Modeling Network; HPV, human papillomavirus; MISCAN, Microsimulation Screening Analysis; NHDS, National Hospital Discharge Survey; SEER, Surveillance, Epidemiology, and End Results Program; UMN-HPV CA, University of Minnesota–HPV Cancer.

^a^ Estimates are for total proportion of cancers by stage that are detected via symptoms (real proportion is lower due to competing causes of death).

^b^ Estimates are for mean duration for proportion of cancers to progress to the next stage.

^c^ Lifetime risks are calculated to age 85 years.

^d^ Estimates are greater than or equal to atypical squamous cells of undetermined significance for detection of CIN of grade 2 or greater.

^e^ Test characteristics of cytology were calibrated to observed data.

^f^ Estimates are high-risk HPV for detection of CIN grade 2 or 3.

^g^ Specificity of clinical HPV testing for CIN grade 2 or 3 was estimated based on (1) the underlying prevalence of specific health states in this modeled population and (2) the test positivity of clinical HPV testing for each of these states as follows: well (no HPV infection) (1.4%), productive HPV infection without CIN grade 1 manifestation (44.0%), and productive HPV infection with CIN grade 1 (84.2%). This yielded an overall specificity of 93.1% for clinical HPV testing for CIN grade 2 or 3.

^h^ Imperfect adherence to colposcopy and colposcopy-directed biopsy (ie, colposcopy and biopsy) and treatment were derived from the Kaiser Permanente Northern California Guidelines cohort using methods based on Cuzick et al^14^ and Kinney et al.^15^ Women aged 21 to 65 years with a baseline screening event of cytology alone between 2006 and 2012 were included; women with hysterectomy, history of CIN grade 2 or 3, or no additional screening within 6 years of baseline were excluded. For colposcopy and biopsy adherence, the proportion of women with a cytology result of atypical squamous cells of undetermined significance, atypical squamous cells that cannot exclude high-grade lesion, low-grade squamous intraepithelial lesion, or high-grade squamous intraepithelial lesion (for women aged 21-29 years) or atypical squamous cells of undetermined significance and HPV positive, atypical squamous cells that cannot exclude high-grade lesion, low-grade squamous intraepithelial lesion, or high-grade squamous intraepithelial lesion (for women aged 30-65 years) who received colposcopy or colposcopically directed biopsy within 1 year of screening was obtained. A weighted average of this proportion across both age groups was calculated. For treatment adherence, the proportion of women receiving excisional or ablative treatment within 1 year of cervical biopsy results of CIN grade 2 or 3 was obtained.

^i^ MISCAN Cervix assumes perfect treatment effects for precancerous lesions through perfect adherence to treatment and treatment efficacy.

An overview of the natural history of cervical cancer represented by the different models is presented in the eFigure in Supplement 1. In the absence of screening, females can progress from HPV infection through CIN to cancer, CIN can regress, and infections can clear naturally. Cancer (squamous cell carcinoma for the Harvard model and all histological types for the other models) can be detected at different stages via symptoms or screening. Females with undetected cancer are at risk of either progressing to the next stage, remaining in the same stage, or dying of cervical cancer. We based cancer survival on age-, stage-, and time-dependent relative survival estimates obtained from the Surveillance, Epidemiology, and End Results Program.^9^ All models account for competing risks of hysterectomy and death from non–cervical cancer causes using age-specific estimates.^10,11,12^ The latter is especially important given the markedly increased risk of competing mortality at older ages. The 4 models simulate current US screening and follow-up recommendations and can accommodate variations in the age to begin and end screening, the screening tests used, screening test sensitivity and specificity, management algorithms based on screening and diagnostic test results, and treatment efficacy.^13^

Key model differences include transition probabilities between health states. One model (Harvard) uses transitions dependent on time spent in a health state, whereas the other 3 models use age-dependent transitions. The models differ in the estimated time between a causal HPV infection and incident cancer overall and by HPV type.^7^ The models also differ in assumptions about the risk of progression between stages of cancer and the stage-specific risk that cancers are detected due to symptoms (Table 1).

### Ascertainment of Outcomes

For comparison with the findings from Landy et al,^6^ the 3- and 5-year risks of CIN3 (or CIN3 or worse [CIN3+] in the MISCAN-Cervix model) in females who met the criteria to exit screening were calculated by assuming a test with perfect sensitivity was applied 3 and 5 years after the age when females met the criteria to exit screening to identify all cases of CIN3 (ie, true health state). For each scenario, we evaluated 2 outcomes: the cumulative risk and the age-conditional risk of cervical cancer incidence and death. The former was chosen to reflect the remaining risk of cancer and cancer death for females who have met the criteria to end screening. The latter was chosen to address concerns that cumulative estimates of risk may obscure differences in risk calculated over a shorter time horizon and to provide estimates for females who have survived to a given age with an intact cervix.^18^

### Data Analysis

#### Comparison With Observed Risk of CIN3

Landy et al^6^ reported the 3- and 5-year risks of CIN3, corrected for unresolved positive test results, among females aged 60 to 64 years who had 2 consecutive negative cotest results as 0.015% (95% CI, 0%-0.031%) and 0.058% (95% CI, 0.006%-0.109%), respectively. These estimates were based on analyses of cervical screening tests (both HPV and cytology) and results as well as colposcopies and biopsies that took place at KPNC between January 1, 2003, and December 31, 2015.^6^ Since the screening history of the females prior to ages 60 to 64 years was not reported in the article, we modeled 4 different scenarios to compare model-estimated 3- and 5-year risks of CIN3 to the empirical study.

Scenario 1 was no screening prior to exit cotests. Females were assumed to have their first screening with cotesting at age 60 years. Females with a negative cotest at age 60 years underwent a second screening at age 63 years (reflecting triennial screening guidelines at KPNC), and if the cotest result was negative, they were classified as having met the criteria to end screening since the next scheduled test would be past age 65 years. Adherence to colposcopy and treatment was assumed to be 100%.

Scenario 2 was a single cotest prior to exit cotests. Females were assumed to begin screening with cotesting at age 57 years to detect and remove prevalent precancers. A subsequent cotest was performed at age 60 years, the result of which was the first used in determining eligibility to end screening. Those with a negative cotest result at age 60 years were retested at age 63 years. Those with a second negative cotest result at age 63 years were classified as having met the US guidelines criteria to exit screening. Adherence to colposcopy and treatment was assumed to be 100%.

Scenario 3 was adhering to US screening guidelines. Females were assumed to adhere to screening with cytology every 3 years beginning at age 21 years and then switching to cotesting every 5 years at age 30 years, consistent with US guidelines. Estimates from the KPNC Guidelines cohort using methods from 2 US studies were used to model nonadherence to colposcopy (78.9%) and treatment (73.4%); results from a meta-analysis informed the estimate of a lower efficacy of treatment (93%).^14,15,19^ Females were assumed to have their first cotest relevant to determining eligibility for screening exit at age 60 years and the second cotest at age 65 years. Females who had a second consecutive negative cotest result at age 65 years were classified as having met the criteria to exit screening.

Scenario 4 was adherence to KPNC screening guidelines. Females were assumed to adhere to screening every 3 years beginning at age 21 years with cytology, with a switch to cotesting every 3 years at age 30 years, to mimic KPNC screening recommendations at the time of the study. As in scenario 3, nonadherence to colposcopy and treatment and lower efficacy of treatment was assumed. Females were assumed to have their first cotest relevant to determining eligibility to end screening at age 60 years, and among those with a negative cotest result, a second cotest was performed at age 63 years to categorize the group as having met the criteria to exit screening.

#### Sensitivity Analyses Using Assumptions for Scenario 1

In 3 models (UMN-HPV CA, Harvard, and Policy1-Cervix), the sensitivity of cytology was decreased to 0.514 (base case, 0.727) to provide an upper bound for cancer and cancer deaths among females only undergoing 2 less sensitive cotests prior to exiting screening.^4,5^ We also conducted a sensitivity analysis using all 4 models in which we increased the incidence of HPV infection by 10% for all ages by applying an assumed relative risk of 1.1 to assess the potential consequences of increased lifetime exposure and/or possible reactivation of HPV infection for cervical cancer incidence and mortality.^20,21^

## Results

### Model Validation to KPNC Data

**Figure 1.**
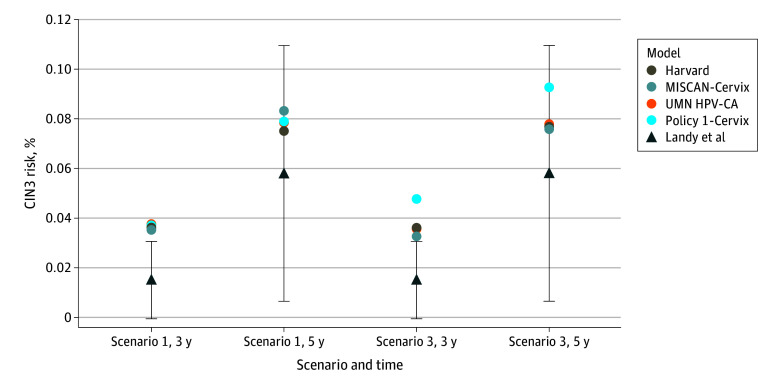
Validation of Risk of Model-Estimated Cervical Intraepithelial Neoplasia Grade 3 (CIN3) Compared With Landy et al^6^ Scenario 1 was no screening prior to age 60 years. Scenario 3 was screening per US guidelines (3-yearly cytology beginning at age 21 years with switch to cotesting every 5 years at age 30 years). Perfect colposcopy sensitivity was assumed for both scenarios. Error bars represent 95% CIs in Landy et al.^6^ MISCAN indicates Microsimulation Screening Analysis and UMN-HPV CA, University of Minnesota–Human Papillomavirus Cancer.

Figure 1 presents a comparison of the 3- and 5-year risks of CIN3 reported by Landy et al^6^ with those estimated by the models for scenario 1 and scenario 3. For scenario 1, the 3-year risks were similar across the 4 models and ranged from 0.035% to 0.038%, exceeding the upper bound of the 95% CI reported by Landy et al.^6^ The 5-year risks estimated by the models were within the 95% CI reported by Landy et al and ranged from 0.075% to 0.083%. For scenario 3, the 3-year risks ranged from 0.032% to 0.048%, again exceeding the 95% CI bounds from Landy et al.^6^ The 5-year risks ranged from 0.076% to 0.092%, falling within the 95% CI bounds of the empirical data. Of note, the lifelong adherence to US screening guidelines in scenario 3 was not consistently associated with lower risks of CIN3 compared with scenario 1; Policy1-Cervix found greater risks of CIN3 in scenario 3, reflecting model differences in natural history by age, long-term vs short-term impact of screening, screening adherence, and age at final screening. Results for scenarios 2 and 4 (eTable 1 in Supplement 1) were similar to those for scenarios 1 and 3.

### Age-Conditional Risks of Cancer and Cancer Death

**Figure 2.**
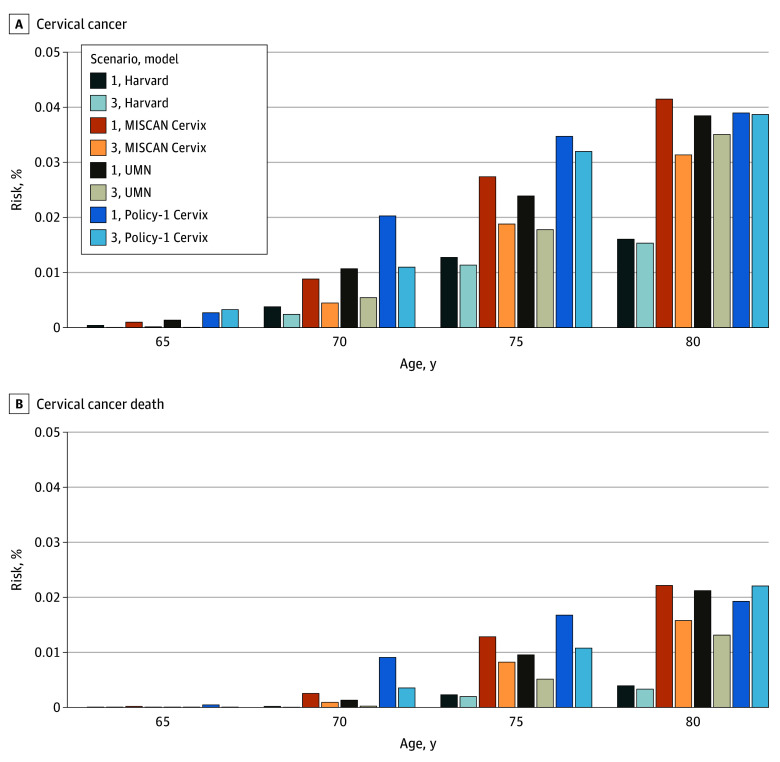
Model-Estimated 5-Year Age-Conditional Risks of Cervical Cancer and Cervical Cancer Death for Scenarios 1 and 3 Age-conditional risks were restricted to females alive with a cervix at age 65, 70, 75, or 80 years. Scenario 1 was no screening prior to age 60 years and scenario 3, screening per US guidelines (3-yearly cytology beginning at age 21 years with switch to cotesting every 5 years at age 30 years). MISCAN indicates Microsimulation Screening Analysis and UMN, University of Minnesota.

Figure 2 presents the 5-year age-conditional risks of cervical cancer and cervical cancer death for scenarios 1 and 3. Although risks for both scenarios were of similar magnitude, lower risks of cervical cancer or cervical cancer death were generally observed among females who had a history of guideline-based screening (scenario 3) compared with females with only 2 consecutive negative cotest results prior to exiting screening (scenario 1). Policy1-Cervix was the exception, estimating a higher age-conditional risk of cervical cancer at age 65 years (0.0033% vs 0.0027%) and a higher age-conditional risk of cervical cancer death at age 80 years (0.022% vs 0.019%) for screened females in scenario 3 compared with scenario 1. Of note, all models estimated that the age-conditional risks of both cervical cancer and cervical cancer death would increase with each 5-year increment in age. Results for scenarios 2 and 4 (eTable 2 in Supplement 1) were similar.

### Cumulative Risk of Cancer and Cancer Death

Table 2 presents the estimated cumulative risks of cervical cancer and cervical cancer death for scenarios 1 and 3. In scenario 1, the estimated cumulative risk of cervical cancer ranged from 0.001% to 0.003% by age 70 years and from 0.026% to 0.081% by age 85 years. The cumulative risk of cervical cancer death ranged from 0% to 0.001% by age 70 years and from 0.005% to 0.038% by age 85 years. For scenario 3, by age 85 years, the estimated risks of cervical cancer and cervical cancer death ranged from 0.023% to 0.077% and from 0.004% to 0.032%, respectively. Results for scenario 2 were similar to those for scenario 1, and results for scenario 4 were similar to those for scenario 3 (eTable 3 in Supplement 1).

**Table 2.** Model-Estimated Cumulative Risk of Cervical Cancer and Cancer Death for Scenarios 1 and 3

Model	Cumulative risk, %							
	Age 70 y		Age 75 y		Age 80 y		Age 85 y	
	Cancer	Cancer death	Cancer	Cancer death	Cancer	Cancer death	Cancer	Cancer death
**Scenario 1^a^**								
Harvard	0.001	0	0.004	0	0.014	0.002	0.026	0.005
MISCAN–Cervix	0.001	0	0.009	0.002	0.030	0.012	0.056	0.026
UMN-HPV CA	0.001	0	0.011	0.001	0.030	0.009	0.056	0.023
Policy1- Cervix	0.003	0.001	0.022	0.009	0.052	0.023	0.081	0.038
**Scenario 3^b^**								
Harvard	0	0	0.002	0	0.012	0.002	0.023	0.004
MISCAN–Cervix	0	0	0.004	0.001	0.019	0.007	0.041	0.018
UMN-HPV CA	0	0	0.005	0	0.019	0.004	0.043	0.014
Policy1- Cervix	0.003	0.003	0.015	0.004	0.045	0.014	0.077	0.032

Abbreviations: MISCAN, Microsimulation Screening Analysis; UMN-HPV CA, University of Minnesota–HPV Cancer.

^a^ No screening prior to age 60 years.

^b^ Screening per US guidelines (3-yearly cytology beginning at age 21 years, with switch to cotesting every 5 years at age 30 years).

When a lower cytology test sensitivity (0.514 [for scenario 1 only]) was assumed, the cumulative risk of cervical cancer by age 85 years was estimated to range from 0.026% to 0.087% (Table 3). The cumulative risk of cervical cancer death by age 85 years was estimated to rise slightly, ranging from 0.005% to 0.041%. In addition, when a 10% increase in HPV incidence across all ages was assumed, the cumulative risk of cervical cancer by age 85 years ranged from 0.027% to 0.093% and the cumulative risk of cervical cancer death ranged from 0.005% to 0.044% (Table 3).

**Table 3.** Sensitivity Analyses of Model-Estimated Cumulative Risk of Cervical Cancer and Cancer Death for Scenario 1

Model	Cumulative risk, %							
	Age 70 y		Age 75 y		Age 80 y		Age 85 y	
	Cancer	Cancer death	Cancer	Cancer death	Cancer	Cancer death	Cancer	Cancer death
**Low cytology sensitivity for CIN2+ (0.514)**								
Harvard	0.001	0	0.004	0.001	0.015	0.002	0.026	0.005
MISCAN–Cervix	NA	NA	NA	NA	NA	NA	NA	NA
UMN-HPV CA	0.002	0	0.011	0.001	0.030	0.009	0.056	0.023
Policy1–Cervix	0.005	0	0.024	0.008	0.059	0.026	0.087	0.041
**10% Increase in human papillomavirus incidence**								
Harvard	0.001	0	0.004	0	0.015	0.002	0.027	0.005
MISCAN – Cervix	0.001	0	0.010	0.002	0.035	0.013	0.063	0.029
UMN-HPV CA	0.001	0	0.011	0.002	0.034	0.011	0.061	0.023
Policy1–Cervix	0.004	0.001	0.026	0.009	0.059	0.025	0.093	0.044

Abbreviations: CIN2+, cervical intraepithelial neoplasia grade 2, grade 3, or cancer; MISCAN, Microsimulation Screening Analysis; NA, not applicable; UMN-HPV CA, University of Minnesota–HPV Cancer.

## Discussion

The optimal age and method for cervical cancer screening cessation remains controversial due to a lack of empirical data to confirm the subsequent risk of cervical cancer and cervical cancer death in females who have fulfilled the criteria to exit screening. A large observational study by Landy et al^6^ did not observe any cancers or cancer deaths during follow-up among females with 2 consecutive negative cotest results, although as the authors noted, the follow-up period may have been insufficient to capture these rare outcomes. We showed that 4 independently developed models of HPV and cervical cancer accurately estimated the 5-year risk of CIN3 among females who met the criteria to exit screening in the US.^6,7^ Across various screening history scenarios, all models estimated a low cumulative risk of subsequently developing or dying from cervical cancer by age 85 years among females with 2 negative cotest results between ages 60 and 65 years, ranging from 0.026% to 0.081% and from 0.005% to 0.038%, respectively. Findings were similar for the age-conditional risks of cancer and cancer death. Both sets of estimates showed that the risks of cancer and cancer death increased with age.

To our knowledge, this is the first study to estimate the age-conditional and cumulative risks of cervical cancer and cervical cancer death for this subset of older, screened females. Two prior US-based analyses of the age to end cervical cancer screening, conducted for the USPSTF, focused on cytology only or cotesting, but for the latter, risk was not quantified separately for the subset of females who met the criteria to exit screening.^4,5^ While the models in the present analysis slightly overestimated the 3-year risk of CIN3 from Landy et al,^6^ all models replicated the 5-year risk of CIN3, which is similar in magnitude to the range in 5-year risk for CIN3+ of 0.029% to 0.046% reported by Castle et al^22^ for a wider age group (≥50 years) of women at KPNC. The slightly higher risks estimated by the models could be due to the assumed perfect ascertainment of CIN3 or the simulation of a cohort rather than the cross-section of females aged 60 to 64 years with different screening histories represented by the empirical data.

The differences in risk observed between the models are primarily due to differences in assumptions regarding the natural history of HPV and cervical cancer.^7,8^ For the Harvard model, which had the lowest risks of cancer and cancer mortality at each age following screening exit, transitions are based on time in state, with persistent infections and lesions at greater risk of progression. For the other 3 models, transitions are based on age (as a surrogate for time in state), with infections and lesions at greater risk of progression at older ages. Of note, the dwell time for HPV infections that cause cancer (ie, causal infections) is longer in the Harvard model than in the other 3 models.^7^ This longer dwell time may allow for HPV-based screening to be more impactful in the Harvard model compared with the other 3 models, leading to greater surveillance and treatment of HPV infections that would otherwise lead to invasive cancer.

Gravitt et al^20,21,23^ have raised concerns about potentially greater HPV exposure in Sexual Revolution birth cohorts that may lead to a corresponding increase in reactivated latent HPV infections at older ages that may not be captured by the HPV prevalence data used to calibrate the models. When we conducted a sensitivity analysis in which we increased the incidence of HPV by an assumed 10% across all ages, cancer incidence increased by 4% to 15%. Going forward, it will be important to discern (1) whether HPV prevalence increases in these incoming cohorts of older women and (2) whether a potential increase in HPV prevalence at these older ages leads to a marked increase in cancer risk.

### Limitations

There are several limitations to this analysis. We validated our modeled estimates of CIN3 against estimates derived from the KPNC population, which may differ from other, more diverse populations in the US. As additional data become available, we will be able to conduct similar modeling validation exercises to ensure that our models accurately estimate the risk of cervical cancer and cervical cancer death for the general US population. We did not evaluate the risks of cervical cancer or cervical cancer mortality under different sets of exit criteria, such as a later age to end screening or screening at age 65 years, when females in the US become eligible for Medicare, as suggested by Dilley et al.^24^ A strategy of a single, standardized exit test may be easier to implement and document than the current recommendation of multiple tests over an extended period.^25^ Our finding of an increasing risk (both cumulative and age-conditional) with increasing age and/or time since last screening, which is consistent with previous analyses,^26^ could potentially be mitigated by a final screening at an older age. However, while prior analyses examining older ages to end screening have suggested benefits in terms of reduced cancer incidence and deaths, the ratio of harms (which may include psychological stress associated with screening, unnecessary surveillance and colposcopies, overtreatment, and excess costs) to benefits also increased, underscoring the need to examine both harms and benefits.^4,5^ In addition, our study was restricted to cervical cancer; comparative modeling studies that quantify the risks and harms over a similar time horizon for females who have met the screening cessation criteria for a range of cancers may also be useful to highlight and address potential inconsistencies in risk thresholds as well as harms-to-benefits ratios.

## Conclusions

In this decision analytical comparative modeling study, 4 independently developed models of cervical cancer estimated low (<0.1% by age 85 years) risks of cancer and cancer death among females who met the current cotesting criteria to exit screening in the US. These results were, however, sensitive to assumptions about HPV incidence (and prevalence), which may increase as Sexual Revolution birth cohorts age, highlighting the need for continued monitoring. While future guidelines could evaluate a greater range of strategies for screening cessation, any policy decision should consider what level of cancer and cancer mortality risk is acceptable. Evaluation of alternative strategies should consider not only the associated cancer and cancer mortality risks but also screening-related harms, taking into account the increasing burden of comorbidities and the competing risk of death in older women.

## Notes

Supplement 1. **eFigure.** Overview of Cervical Cancer Model Natural History States and Allowed Transitions **eTable 1.** Model-Estimated 3- and 5-Year Risk of CIN3 for Scenarios 2 and 4 **eTable 2.** Model-Estimated Age-Conditional Risks of Cervical Cancer and Cancer Death for Scenarios 2 and 4 **eTable 3.** Model-Estimated Cumulative Risks of Cervical Cancer and Cancer Death for Scenarios 2 and 4

Supplement 2.
Data Sharing Statement
