# Supplementary material for: Estimated Cancer Risk in Females Who Meet the Criteria to Exit Cervical Cancer Screening
Source: JAMA Netw Open. 2025 Mar 12;8(3):e250479. doi: 10.1001/jamanetworkopen.2025.0479 (PMC11904717; doi:10.1001/jamanetworkopen.2025.0479)
Supplement: Supplement 1. — eFigure. Overview of Cervical Cancer Model Natural History States and Allowed Transitions eTable 1. Model-Estimated 3- and 5-Year Risk of CIN3 for Scenarios 2 and 4 eTable 2. Model-Estimated Age-Conditional Risks of Cervical Cancer and Cancer Death for Scenarios 2 and 4 eTable 3. Model-Estimated Cumulative Risks of Cervical Cancer and Cancer Death for Scenarios 2 and 4 [file jamanetwopen-e250479-s001.pdf]

Supplementary Online Content

Kulasingam SL, de Kok IMCM, Mehta A, et al. Estimated cancer risk in females who meet the criteria to exit cervical cancer screening. *JAMA Netw Open*. 2025;8(3):e250479. doi:10.1001/jamanetworkopen.2025.0479

eFigure. Overview of Cervical Cancer Model Natural History States and Allowed Transitions
eTable 1. Model-Estimated 3- and 5-Year Risk of CIN3 for Scenarios 2 and 4
eTable 2. Model-Estimated Age-Conditional Risks of Cervical Cancer and Cancer Death for Scenarios 2 and 4
eTable 3. Model-Estimated Cumulative Risks of Cervical Cancer and Cancer Death for Scenarios 2 and 4

This supplementary material has been provided by the authors to give readers additional information about their work.

© 2025 Kulasingam SL et al. *JAMA Network Open*.

eFigure. Overview of cervical cancer model natural history states and allowed transitions.

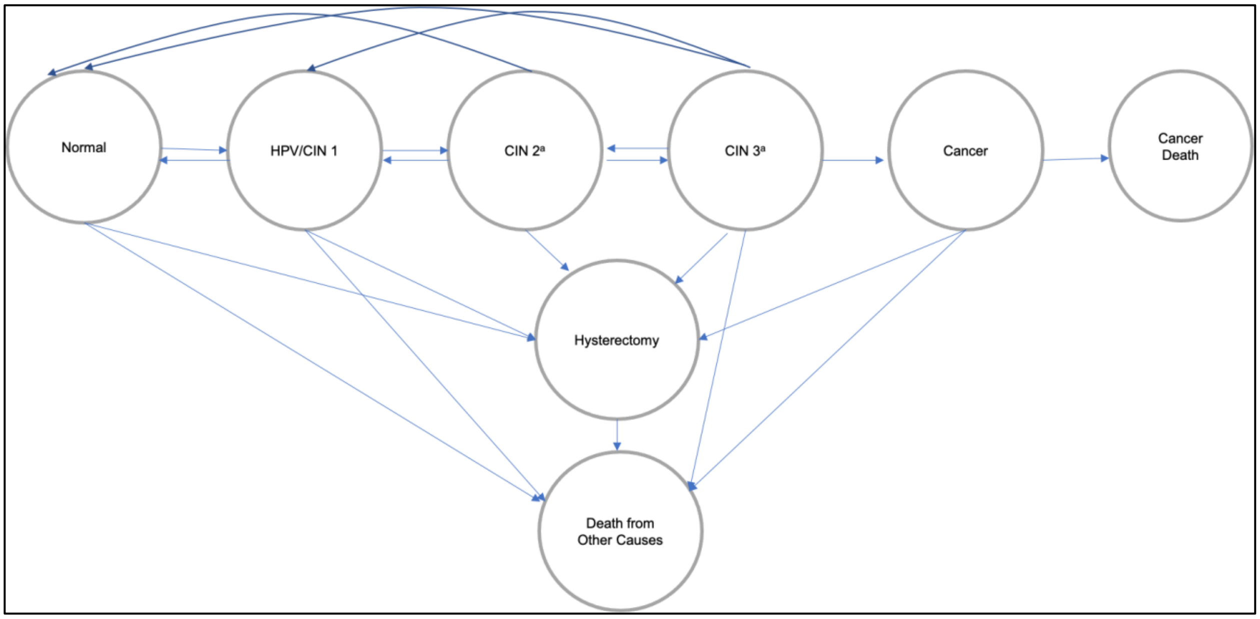

[a] The Harvard model does not contain a health state for cervical intraepithelial neoplasia (CIN) 1, which is considered to be a microscopic manifestation of acute HPV infection and thus incorporated into the HPV state. The Harvard model also considers CIN2 and CIN3 as nonsequential health states with distinct transitions to cancer.

© 2025 Kulasingam SL et al. *JAMA Network Open*.

eTable 1. Model-estimated 3- and 5-year risk of CIN3 for Scenarios 2[a] and 4[b]

	Scenario 2[a]		Scenario 4[b]	
	3 Year Risk	5 Year Risk	3 Year Risk	5 Year Risk
**Harvard**	0.031%	0.072%	0.034%	0.075%
**MISCAN – Cervix**	0.035%	0.084%	0.034%	0.082%
**UMN-HPV CA**	0.031%	0.067%	0.033%	0.063%
**Policy1- Cervix**	0.039%	0.082%	0.038%	0.081%

[a]2 – Single cotest prior to exit cotests
[b]4 – Screening according to Kaiser Permanente Northern California guidelines

© 2025 Kulasingam SL et al. *JAMA Network Open*.

eTable 2. Model-estimated age-conditional risks of cervical cancer and cancer death for Scenarios 2[a] and 4[b].

	Age 65		Age 70		Age 75		Age 80	
	CA	CA death	CA	CA death	CA	CA death	CA	CA death
**Scenario 2[a]**								
**Harvard**	0.000%	0.000%	0.003%	0.000%	0.014%	0.002%	0.016%	0.004%
**MISCAN Cervix**	0.001%	0.000%	0.009%	0.002%	0.026%	0.012%	0.048%	0.023%
**UMN**	0.001%	0.000%	0.010%	0.001%	0.023%	0.008%	0.036%	0.016%
**Policy-1 Cervix**	0.003%	0.000%	0.019%	0.006%	0.033%	0.016%	0.037%	0.020%
**Scenario 4[b]**								
**Harvard**	0.000%	0.000%	0.003%	0.000%	0.014%	0.001%	0.016%	0.005%
**MISCAN Cervix**	0.001%	0.000%	0.009%	0.003%	0.027%	0.013%	0.042%	0.022%
**UMN**	0.000%	0.000%	0.010%	0.001%	0.023%	0.007%	0.037%	0.019%
**Policy-1 Cervix**	0.001%	0.000%	0.019%	0.007%	0.037%	0.016%	0.037%	0.022%

[a]2 – Single cotest prior to exit cotests
[b]4 – Screening according to Kaiser Permanente Northern California guidelines

© 2025 Kulasingam SL et al. *JAMA Network Open*.

eTable 3. Model-estimated cumulative risks of cervical cancer and cancer death for Scenarios 2[a] and 4[b].

	**Age 70**		**Age 75**		**Age 80**		**Age 85**	
	CA	CA death	CA	CA death	CA	CA death	CA	CA death
**Scenario 2[a]**								
**Harvard**	0.000%	0.000%	0.003%	0.000%	0.015%	0.002%	0.026%	0.004%
**MISCAN – Cervix**	0.001%	0.000%	0.009%	0.002%	0.029%	0.012%	0.059%	0.026%
**UMN-HPV CA**	0.001%	0.000%	0.009%	0.001%	0.028%	0.007%	0.052%	0.018%
**Policy1- Cervix**	0.003%	0.000%	0.021%	0.006%	0.049%	0.020%	0.078%	0.035%
**Scenario 4[b]**								
**Harvard**	0.000%	0.000%	0.003%	0.000%	0.014%	0.001%	0.026%	0.005%
**MISCAN – Cervix**	0.001%	0.000%	0.009%	0.002%	0.030%	0.012%	0.056%	0.026%
**UMN-HPV CA**	0.000%	0.000%	0.009%	0.001%	0.027%	0.007%	0.052%	0.020%
**Policy1- Cervix**	0.002%	0.001%	0.021%	0.007%	0.055%	0.021%	0.085%	0.039%

[a]2 – Single cotest prior to exit cotests
[b]4 – Screening according to Kaiser Permanente Northern California guidelines

© 2025 Kulasingam SL et al. *JAMA Network Open*.
