# Supplementary material for: Estimated Cancer Risk in Females Who Meet the Criteria to Exit Cervical Cancer Screening
Source: JAMA Netw Open. 2025 Mar 12;8(3):e250479. doi: 10.1001/jamanetworkopen.2025.0479 (PMC11904717; doi:10.1001/jamanetworkopen.2025.0479)
Supplement: Supplement 2. — Data Sharing Statement [file jamanetwopen-e250479-s002.pdf]

Data Sharing Statement

Kulasingam. Estimated Cancer Risk in Females Who Meet the Criteria to Exit Cervical Cancer Screening. *JAMA Netw Open*. Published March 12, 2025.
doi:10.1001/jamanetworkopen.2025.0479

Data
Data available: No

Additional Information
Explanation for why data not available: The data for this study are detailed in Table 1, the Supplement and published articles detailing the 4 models used for this analysis. Further details are available from the corresponding author (email: kulas016@umn.edu)
